# A CMOS Capacitive Fingerprint Sensor Chip with Column-Shared Charge-Accumulation Readout for Ridge–Valley Contrast Enhancement

**DOI:** 10.3390/s26196335

**Published:** 2026-10-08

**Authors:** Kyungsaeng Kim

**Affiliations:** Department of System Semiconductor Engineering, Cheongju University, Cheongju 28503, Republic of Korea; kyungsaengkim@cju.ac.kr

**Keywords:** capacitive fingerprint sensor, CMOS sensor chip, charge-accumulation readout, column-shared integrator, ridge–valley contrast, image-based ridge–valley SNR, fingerprint image quality, biometric sensor

## Abstract

**Highlights:**

**What are the main findings?**

- A 0.18 μm CMOS capacitive fingerprint sensor chip with a column-shared charge-accumulation readout was implemented and experimentally evaluated.
- For the nine retained frames per accumulation setting, the average ridge–valley contrast increased from 5.71% at *N* = 1 to 43.86% at *N* = 5, while the average image-based ridge–valley SNR remained above 12 dB.

**What are the implications of the main findings?**

- The proposed readout enhances ridge–valley separability without increasing pixel-level circuit complexity.
- The accumulation number *N* should be selected by considering the trade-off among ridge–valley contrast, capture time, frame energy, active sensing current, and dynamic range.

**Abstract:**

Capacitive fingerprint sensors are widely used in mobile, access-control, IoT, and biometric authentication systems because of their compact size, low power consumption, low cost, and CMOS compatibility. However, the small capacitance difference between fingerprint ridges and valleys can be degraded by passivation thickness, skin condition, contact pressure, contamination, and circuit noise, especially in high-resolution sensors with reduced pixel pitch. This work presents a CMOS capacitive fingerprint sensor chip with a column-shared charge-accumulation readout to enhance ridge–valley contrast. The proposed readout repeatedly senses the same pixel and accumulates the resulting charge-domain signals at the column level before analog-to-digital conversion, improving ridge–valley separability without increasing pixel-level circuit complexity. The chip was fabricated in a 0.18 μm CMOS process and includes a 144 × 40 sensor array with a 50 μm pixel pitch, corresponding to approximately 508 dpi. Fingerprint images were acquired from two participants for accumulation numbers from *N* = 1 to *N* = 5. After image-quality screening, the average ridge–valley contrast increased from 5.71% to 43.86%, corresponding to a contrast ratio of approximately 7.68, while the average image-based ridge–valley SNR remained above 12 dB. The Energy-Contrast Efficiency (ECE) increased with *N*, although capture time and frame energy also increased. These results indicate that the proposed readout enhances ridge–valley contrast while the image-based ridge–valley SNR remains relatively stable.

## 1. Introduction

Biometric authentication has become increasingly important in mobile devices, access-control systems, Internet-of-Things (IoT) devices, and embedded security systems. Among various biometric modalities, fingerprint recognition is one of the most widely used approaches because it provides a favorable balance between reliability, convenience, cost, and system-level integration [1]. In particular, CMOS capacitive fingerprint sensors can detect the spatial distribution of fingerprint patterns by sensing capacitance variations between the sensor electrodes and the finger surface, without requiring bulky optical components. Therefore, they are attractive for compact, low-power, and low-cost biometric sensor systems [1,2,3,4].

In a capacitive fingerprint sensor, the effective capacitance between the sensor plate and the finger depends on the local distance and dielectric environment. When a finger is placed on the sensor surface, ridge regions are located closer to the sensing electrodes and produce larger effective capacitance, whereas valley regions include a larger air gap and produce smaller effective capacitance [3,5]. The resulting ridge–valley capacitance difference is converted into an electrical signal by the readout circuit and then digitized to reconstruct the fingerprint image.

However, the ridge–valley capacitance difference in capacitive fingerprint sensors is typically very small. It can be further affected by passivation thickness, skin condition, moisture, contamination, contact pressure, and circuit noise [2,6,7,8]. This problem becomes more critical in high-resolution sensors because the reduced pixel pitch decreases the sensing capacitance per pixel, which in turn reduces the available signal swing for a given capacitance difference [3,7,9]. As a result, a single sensing operation may not provide sufficient ridge–valley contrast, especially under nonideal contact or packaging conditions.

Several circuit and system-level techniques have been reported to improve the sensitivity and image quality of capacitive fingerprint sensors. Differential sensing, charge-transfer sensing, feedback capacitive sensing, capacitive-sensing circuit techniques, correlated double sampling (CDS), baseline subtraction, and low-noise analog front-end circuits have been used to reduce offset, parasitic effects, low-frequency noise, and environmental disturbance [2,3,4,5,6,10]. More recently, low-noise front-end circuits and capacitive readout techniques have been investigated for under-glass or extended sensing-distance applications, where the capacitance signal is even smaller and the sensing range becomes a critical design constraint [7,11,12,13]. These approaches improve the stability and sensitivity of the readout path. Nevertheless, when the signal swing generated by the ridge–valley capacitance difference itself is small, offset cancellation and noise reduction alone may not be sufficient to substantially enhance the visible ridge–valley contrast.

To address this issue, this work presents a CMOS capacitive fingerprint sensor chip with a column-shared charge-accumulation readout for ridge–valley contrast enhancement. The proposed readout repeatedly senses the same pixel and accumulates the resulting charge-domain signals at the column level before analog-to-digital conversion. By sharing the charge-accumulation integrator at the column level, the proposed structure enhances ridge–valley separability without increasing pixel-level circuit complexity. The main objective of this work is not to claim direct recognition-rate improvement, but to demonstrate recognition-relevant image-quality enhancement through improved ridge–valley contrast and relatively stable image-based ridge–valley SNR.

The main contributions of this work are summarized as follows. First, a column-shared charge-accumulation readout architecture is proposed for CMOS capacitive fingerprint sensors to enhance the signal separation between fingerprint ridges and valleys. Second, the proposed readout accumulates charge-domain signals at the column level, thereby avoiding additional circuit complexity in each pixel. Third, a 0.18 μm CMOS capacitive fingerprint sensor chip with a 144 × 40 sensor array, 50 μm pixel pitch, and approximately 508 dpi resolution was implemented and experimentally evaluated. Fourth, fingerprint images were acquired for accumulation numbers from *N* = 1 to *N* = 5 and analyzed using the same region of interest and ridge/valley classification criteria. Finally, the nine-frame results after screening show that the average ridge–valley contrast increased from 5.71% at *N* = 1 to 43.86% at *N* = 5, corresponding to a contrast ratio of approximately 7.68, while the average image-based ridge–valley SNR remained above 12 dB for all accumulation numbers.

## 2. Materials and Methods

This section describes the proposed CMOS capacitive fingerprint sensor chip, including the overall readout architecture, capacitive fingerprint sensing principle, sensing cell front-end, column-shared charge-accumulation readout, timing sequence, signal accumulation model, image-quality metrics, chip implementation, and measurement setup. The proposed readout scheme enhances ridge–valley contrast by repeatedly sensing the same pixel and accumulating the resulting charge-domain signals at the column level before analog-to-digital conversion.

### 2.1. Proposed Circuit Architecture

**Figure 1:**
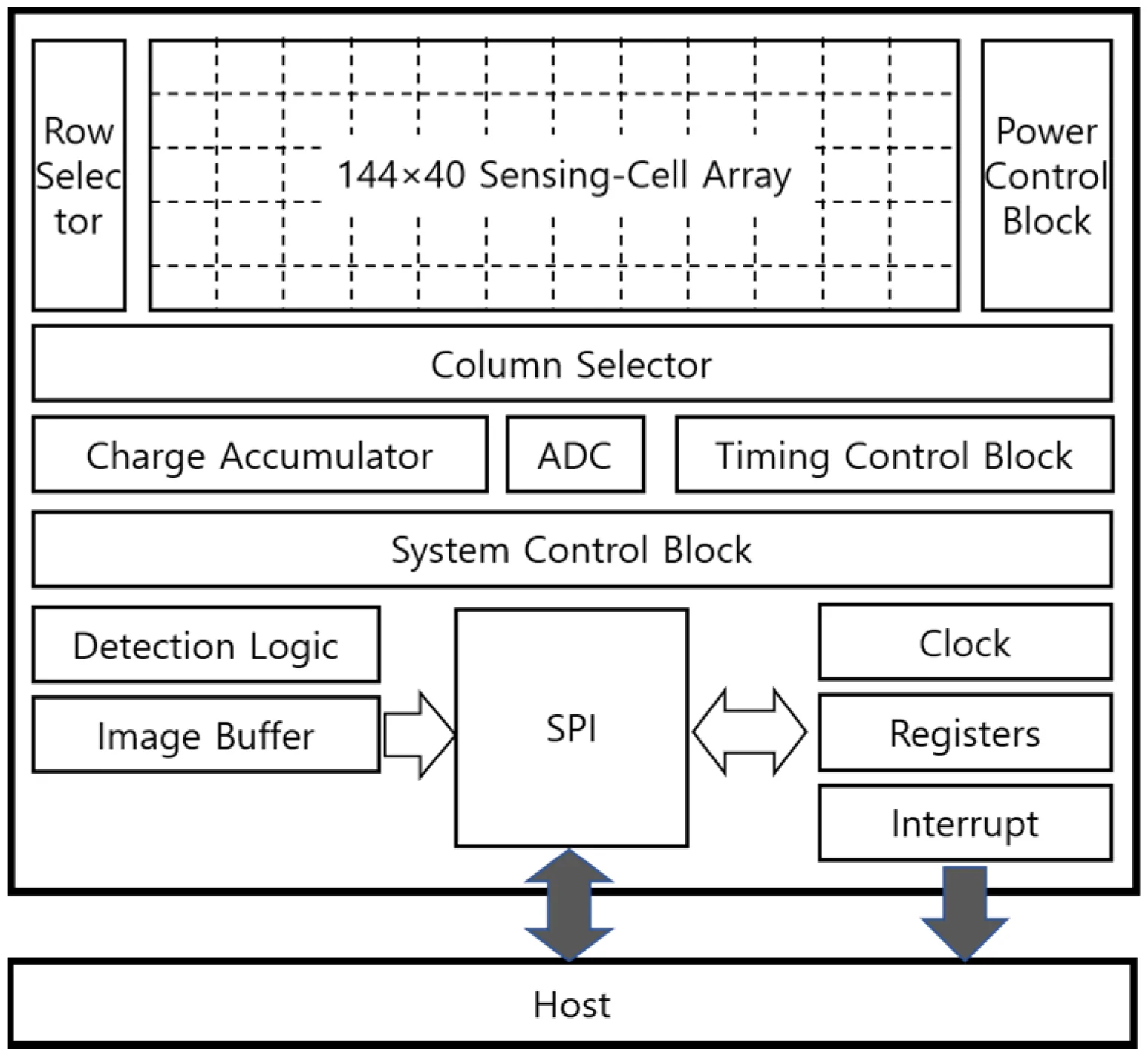
Overall block diagram of the proposed CMOS capacitive fingerprint sensor chip. Gray arrows indicate external connections between the chip and the host, whereas white arrows indicate internal connections between circuit blocks.

The proposed CMOS capacitive fingerprint sensor chip consists of a 144 × 40 capacitive sensing-cell array, row and column selection circuits, a column-shared charge-accumulation integrator, an analog-to-digital converter (ADC), a timing control block, a power control block, an image buffer, a serial peripheral interface (SPI), and a system control block, as shown in Figure 1. In each sensing cell, the local capacitance between the finger and the sensor plate varies with the ridge–valley pattern, and the resulting capacitance variation is converted into an electrical signal by the sensing circuit [3,5].

The signals from the selected row are transferred through the column readout paths to the column-shared charge-accumulation integrator. The accumulated analog output is then digitized by the ADC and stored in the image buffer or transferred to the host through the SPI. This system-level organization is consistent with conventional CMOS capacitive fingerprint sensor implementations, in which the sensing array, selection circuits, analog front-end, ADC, and digital interface are integrated on a single chip [3,11,14].

The key feature of the proposed architecture is repeated sensing of the same pixel, followed by column-level accumulation of the charge-domain signal generated during each sensing operation. In a switched-capacitor charge integrator, the sensed charge can be accumulated by retaining the charge stored on the feedback or accumulation capacitor during repeated sensing cycles [11]. In this work, this charge-retention principle is implemented using a column-shared charge-accumulation integrator. Consequently, the output signal difference between ridge and valley regions can be increased without increasing pixel-level circuit complexity.

### 2.2. Capacitive Fingerprint Sensing Principle

**Figure 2:**
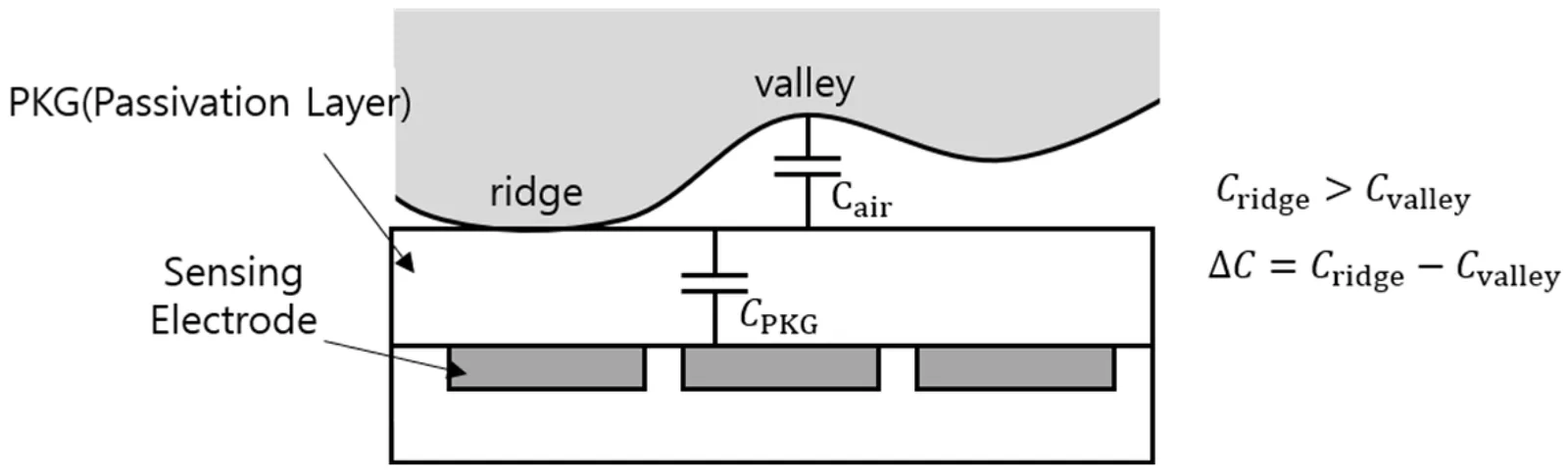
Conceptual cross-sectional view of capacitive fingerprint sensing and the effective capacitance difference Δ*C* between ridge and valley regions.

In a capacitive fingerprint sensor, the effective capacitance between the sensor electrode and the finger surface is determined by the electrode area, passivation thickness, dielectric properties, air gap, and contact condition [2,3,4,5]. When a finger is placed on the sensing surface, ridge regions are closer to the sensor electrodes and therefore form a larger effective capacitance. In contrast, valley regions include a larger air gap and form a smaller effective capacitance [3,4]. The readout circuit converts this spatial capacitance difference into an electrical signal that can be digitized and reconstructed as a fingerprint image. Figure 2 illustrates the principle of capacitive fingerprint sensing and the effective capacitance difference between ridge and valley regions.

Using a simplified parallel-plate approximation, the effective capacitance can be expressed as

(1) $$C=\frac{\varepsilon_{0}\varepsilon_{r}A}{d}$$

where *ε*_0_ is the vacuum permittivity, *ε_r_* is the effective relative permittivity, *A* is the sensing electrode area, and *d* is the effective distance between the sensing electrode and the finger surface. The capacitance difference between ridge and valley regions is defined as

Δ*C* = *C*_ridge_ − *C*_valley_ (2)

where *C*_ridge_ and *C*_valley_ denote the effective capacitances of the ridge and valley regions, respectively.

In practical capacitive fingerprint sensors, the effective ridge–valley capacitance difference Δ*C* can be very small when the package and passivation layers are relatively thick. Because these layers form a large common part of the effective sensing distance in both ridge and valley regions, the additional air gap in the valley region produces only a small change in the total effective distance. Consequently, *C*_ridge_ remains larger than *C*_valley_, but the difference Δ*C* = *C*_ridge_ − *C*_valley_ can remain in the sub-femtofarad range. Therefore, the readout circuit must convert this small capacitance difference into an electrical signal that provides sufficiently distinguishable ridge–valley image contrast.

Fingerprint image quality is closely related to ridge strength, ridge continuity, ridge clarity, and ridge–valley structure integrity, which are important for minutiae extraction and verification performance [8,15]. Therefore, this work quantitatively evaluates the measured fingerprint images using ridge–valley contrast and image-based ridge–valley SNR under the same region of interest and the same ridge/valley classification criteria.

### 2.3. Sensing Cell Front-End

**Figure 3:**
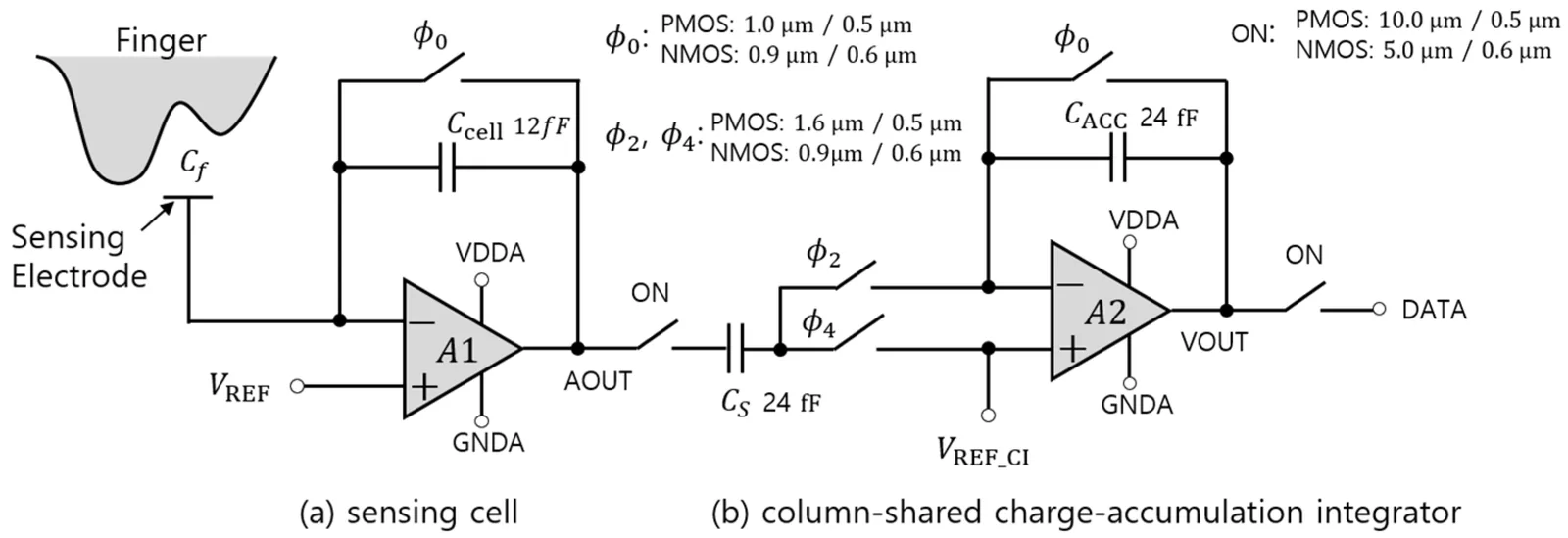
Proposed fingerprint sensing readout architecture: (**a**) sensing cell front-end and (**b**) column-shared charge-accumulation integrator. The capacitor values, switch transistor dimensions, and main switch-control signals are indicated.

The sensing cell front-end converts the capacitance variation induced by fingerprint ridges and valleys into a charge-domain signal. As shown in Figure 3a, the sensing cell consists of a sensing electrode, a cell capacitor *C*_cell_, a switch network, and an operational transconductance amplifier (OTA). During the sampling phase, the capacitance-dependent signal at the sensing electrode is sampled onto *C*_cell_ relative to a reference voltage. The corresponding charge variation produces a voltage change at AOUT, which is sampled by the sampling capacitor in the following column readout path.

The charge variation generated by the ridge–valley capacitance difference can be expressed as

Δ*Q*_cell_ = Δ*C*·Δ*V* (3)

where Δ*Q*_cell_ is the signal-charge variation generated in the sensing cell by the ridge–valley capacitance difference, Δ*C* is the effective capacitance difference between ridge and valley regions, and Δ*V* is the effective voltage difference formed between the sensing electrode and the finger surface. In capacitive readout circuits, this charge-domain signal can be converted into a voltage-domain signal through charge sampling and redistribution using switched-capacitor elements, such as the cell capacitor and the feedback or accumulation capacitor [11].

In the proposed sensing cell, the reset, sampling, and baseline-subtraction operations are intended to provide a CDS-like sampled-data function for reducing common offset components and low-frequency noise components, including 1/f noise. The *ϕ*_0_-controlled reset operation initializes the sensing and accumulation nodes before charge-domain signal sampling. Such sampled-data techniques are widely used in capacitive readout circuits to reduce offset and low-frequency noise effects [10]. However, the proposed circuit does not employ a separate dedicated CDS amplifier or a complete self-zeroing circuit. Therefore, residual DC offset, 1/f noise, switch charge injection, and clock feedthrough may not be completely eliminated and may still affect the charge accumulation process.

**Figure 4:**
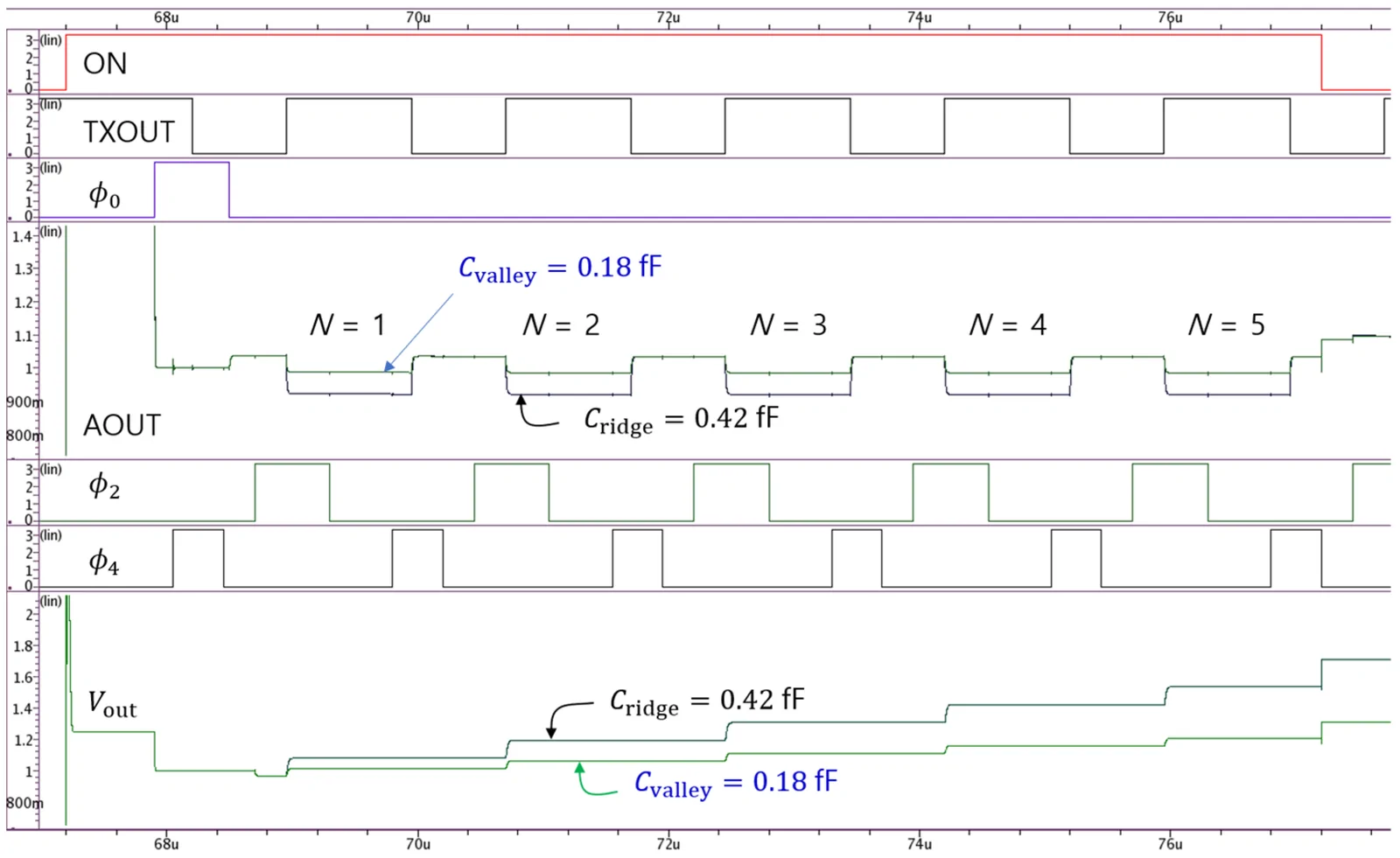
Simulated transient waveforms of the proposed reset, sampling, accumulation, and hold operations for an accumulation number of *N* = 5.

The main switch-control signals are indicated in Figure 3, and the corresponding simulated TXOUT waveform is shown in Figure 4. TXOUT is an internal sensing-control clock, and the sensing operation is synchronized with this signal. The *ϕ*_0_ signal is used for the reset operation. When *ϕ*_0_ is high, the sensing cell and the column-shared charge-accumulation integrator are reset. After *ϕ*_0_ becomes low, the internal TXOUT signal defines the reference and sensing conditions of the sensing cell. When TXOUT is low, the reference finger-capacitance condition is sampled. When TXOUT is high, the capacitance variation associated with the local ridge or valley condition, relative to the reference condition, is sampled as a charge-domain difference signal. The *ϕ*_4_ phase connects the integrator-side plate of *C*_S_ to *V*_REF_CI_ for sampling, whereas *ϕ*_2_ connects that plate to the summing node of A2 for charge transfer. The capacitor values and the PMOS/NMOS dimensions (W/L) of the switches controlled by *ϕ*_0_, *ϕ*_2_, *ϕ*_4_, and ON are indicated in Figure 3. In pre-layout circuit simulations at a 3.3 V supply, the OTA exhibits a DC open-loop gain of approximately 40 dB and a unity-gain bandwidth of approximately 36.3 MHz. The OTAs used in the sensing and charge-accumulation circuits employ the same circuit topology and have the same nominal simulated analog characteristics. A separate quantitative characterization of the OTA noise is not reported in this work.

### 2.4. Column-Shared Charge-Accumulation Readout

The charge-domain output of the sensing cell is proportional to the effective ridge–valley capacitance difference Δ*C*. However, because Δ*C* is very small in capacitive fingerprint sensing, a single sensing operation may not provide sufficient signal separation between ridge and valley regions [2,3,5,6,7]. To enhance ridge–valley separability, the proposed readout repeatedly senses the same pixel and accumulates the resulting Δ*C*-dependent charge-domain signal in a column-shared charge-accumulation integrator.

As shown in Figure 3b, the column-shared charge-accumulation integrator consists of a sampling capacitor, an accumulation capacitor *C*_ACC_, an OTA, and a switch network driven by non-overlapping clock phases. During each sensing operation for the same selected pixel, the AOUT variation is sampled by *C_S_*, and the corresponding sampled charge is transferred to *C*_ACC_. The transferred charge is stored on *C*_ACC_. Because *C*_ACC_ is not reset between repeated sensing cycles, the newly transferred charge is added to the charge already stored on *C*_ACC_. As a result, the ridge–valley charge-domain signal is accumulated as the accumulation number *N* increases. This charge-retention operation at the column level enables repeated accumulation across sensing cycles without adding an accumulation circuit inside each pixel.

The output voltage change generated by a single sensing operation can be approximated as

(4) $$\Delta V_{\mathrm{out},1}\approx \frac{\Delta Q_{S}}{C_{\mathrm{ACC}}}=\frac{C_{S}}{C_{\mathrm{ACC}}}\Delta V_{\mathrm{AOUT}}$$

where Δ*Q_S_* is the signal-charge increment transferred from the sampling capacitor *C_S_* to the accumulation capacitor *C*_ACC_ in one sensing cycle, and Δ*V*_AOUT_ is the corresponding voltage change at AOUT. Under ideal charge transfer, Δ*Q_S_* = *C_S_*Δ*V*_AOUT_. For an ideally settled sensing front-end, Δ*V*_AOUT_ ≈ Δ*Q*_cell_/*C*_cell_, where Δ*Q*_cell_ is the signal-charge variation defined in Equation (3). These relationships describe signal magnitudes.

When the same pixel is sensed *N* times and the charge contributions from the repeated sensing operations are ideally accumulated, the output voltage change after *N* accumulation cycles can be expressed as

(5) $$\Delta V_{\mathrm{out},N}=N\Delta V_{\mathrm{out},1}\approx \frac{N\Delta Q_{S}}{C_{\mathrm{ACC}}}=N\frac{C_{S}}{C_{\mathrm{ACC}}}\Delta V_{\mathrm{AOUT}}$$

Equation (5) indicates that the proposed column-shared readout increases the output signal difference between ridge and valley regions by accumulating the small charge-domain signals generated in repeated sensing cycles. Because the accumulation operation is performed at the column level rather than inside each pixel, the proposed architecture can enhance ridge–valley contrast while avoiding additional pixel-level circuit complexity.

However, the accumulation number *N* cannot be increased indefinitely. As *N* increases, the output swing of the column-shared charge-accumulation integrator also increases. If the accumulated output exceeds the sensing range of the readout circuit or the input range of the ADC, output saturation or charge overflow can occur [12]. Therefore, *N* should be selected such that the accumulated output remains within the allowable dynamic range.

The saturation-avoidance condition can be expressed as

(6) $$V_{\mathrm{out},0}+N{\left|\Delta V_{\mathrm{out},1}\right|}_{\max}<V_{\max}$$

where *V*_out,0_ is the initial output voltage, *V*_max_ is the maximum allowable output voltage before op-amp output saturation or ADC input-range limitation, and ${\left|\Delta V_{\mathrm{out},1}\right|}_{\max}$ is the maximum magnitude of the single-cycle output change caused by the largest expected capacitance-dependent charge variation.

The maximum signal-charge increment transferred from *C_S_* to *C*_ACC_ in one sensing cycle is denoted by Δ*Q*_S,max_. Under the ideal settling and charge-transfer assumptions used in Equation (4), it can be approximated as

$$\Delta Q_{S,\max}\approx \frac{C_{S}}{C_{\mathrm{cell}}}{\left|\Delta C\right|}_{\max}\Delta V_{S}$$

where |Δ*C*|_max_ is the largest expected magnitude of the effective capacitance variation between the reference condition and the local ridge or valley sensing condition, and Δ*V*_S_ is the effective sensing voltage swing.

The corresponding maximum magnitude of the single-cycle output change is then

$${\left|\Delta V_{\mathrm{out},1}\right|}_{\max}\approx \frac{\Delta Q_{S,\max}}{C_{\mathrm{ACC}}}$$

Substituting this relation into the saturation-avoidance condition in Equation (6), the maximum admissible integer accumulation number is

(7) $$N_{\max,\mathrm{theory}}=\left\lceil \frac{V_{\max}-V_{\mathrm{out},0}}{{\left|\Delta V_{\mathrm{out},1}\right|}_{\max}}\right\rceil -1=\left\lceil \frac{\left(V_{\max}-V_{\mathrm{out},0}\right)C_{\mathrm{ACC}}}{\Delta Q_{S,\max}}\right\rceil -1$$

For fixed values of the other parameters, Equation (7) shows that *N*_max,theory_ increases with the available output swing and *C*_ACC_, but decreases as Δ*Q*_S,max_ increases. The ceiling-minus-one form enforces the strict inequality in Equation (6), including the case in which the ratio is an integer.

### 2.5. Timing Sequence

The proposed readout operation consists of reset, sampling, accumulation, and hold phases. During the reset phase, the accumulation capacitor is initialized to the reference voltage. During the sampling phase, the signal from the selected sensing cell is sampled onto the sampling capacitor. During the accumulation phase, the sampled charge is transferred to the accumulation capacitor, resulting in a stepwise change in the output voltage of the column-shared charge-accumulation integrator. After the repeated accumulation cycles are completed, the hold phase maintains the accumulated analog output for analog-to-digital conversion.

Figure 4 shows the simulated transient waveforms of the proposed readout operation for an accumulation number of *N* = 5. The sampling and charge-transfer/accumulation phases are driven by non-overlapping clock signals to reduce the effects of switch charge injection and clock feedthrough. However, these switching-related error components may not be completely eliminated and can appear as residual offset-like charge errors during repeated accumulation. For each selected pixel, the sensing, sampling, and charge-transfer/accumulation operations are repeated according to the accumulation number *N*. The stepwise increase in *V*_out_ shown in Figure 4 illustrates that the sampled charge difference is repeatedly transferred to and retained on *C*_ACC_ across multiple sensing cycles. As *N* increases, the accumulated output difference between ridge and valley regions increases, which can enhance ridge–valley contrast. However, increasing *N* also increases the capture time and frame energy because additional sensing and accumulation cycles are required.

From the representative simulated transient waveform shown in Figure 4, *V*_out,0_ is approximately 1.0 V and the representative single-cycle output increment is approximately 0.1 V. Considering process variation and the linear operating ranges of the readout amplifiers and the ADC input stage, *V*_max_ is assumed to be 3.0 V for the accumulation-limit estimate. Under these assumptions, the strict inequality in Equation (6) gives *N* < 20, with 19 as the largest integer accumulation number below the assumed output limit. This is a model-based operating-limit estimate, not a measured clipping threshold.

The fabricated chip was designed to support accumulation numbers from *N* = 1 to *N* = 5. This range was selected to enlarge weak ridge–valley signals within the intended SPI-synchronous operating conditions, rather than to operate near the analog saturation limit. The measurements covered the full implemented accumulation range. Because *N* > 5 is not supported by the present implementation, an accumulation-number sweep to the extrapolated output limit could not be performed.

The accumulated analog output is converted by the ADC after the final accumulation cycle. Therefore, multiple sensing operations are performed and their charge-domain signals are accumulated before a single ADC conversion, improving ridge–valley separability without requiring an ADC conversion after each sensing cycle.

### 2.6. Signal Accumulation Model and Image-Quality Metrics

Let *S*_1_ denote the signal component obtained from a single sensing operation. When the same pixel is sensed *N* times and the signal components are ideally accumulated with the same polarity, the accumulated signal *S_N_* can be expressed as

*S_N_* = *NS*_1_ (8)

This relationship indicates that repeated charge accumulation can increase the average signal difference between ridge and valley regions.

The equivalent output noise of a single sensing operation includes thermal noise and 1/f noise of the OTA, wideband noise aliasing caused by sampling, switch charge injection, clock feedthrough, and other nonidealities in switched-capacitor circuits [10]. In addition, environmental noise and readout-circuit noise can degrade the circuit-level SNR of capacitive readout systems [11,12]. If the noise generated in each sensing operation is assumed to have zero mean, to be mutually uncorrelated, and to have the same variance, the noise variance after *N* accumulation cycles becomes $N\sigma_{1}^{2}$. Therefore, the rms noise after *N* accumulation cycles can be expressed as

(9) $$\sigma_{N}=\sqrt{N}\cdot \sigma_{1}$$

where *σ*_1_ is the rms noise of a single sensing operation.

More generally, for random cycle errors *n*_i_, the variance of their accumulated sum is

(9a) $$\sigma_{N}^{2}=\sum_{i=1}^{N}\sigma_{i}^{2}+2\sum_{1\leq i<j\leq N}\mathrm{Cov}\left(n_{i},\;n_{j}\right)$$

where $\sigma_{i}^{2}$ is the variance in cycle *i*. Equation (9) is recovered when the covariance terms vanish and the cycle variances are equal. A nonzero per-cycle mean error, if repeated across cycles, accumulates coherently and is not captured by the variance terms above. These assumptions were not independently verified experimentally, so Equation (9) remains an ideal reference model rather than a measured noise law.

Under the idealized first-order assumption that the signal components are linearly accumulated while the noise components are uncorrelated, the circuit-level SNR after *N* accumulation cycles is given by

(10) $${\mathrm{SNR}}_{N}=\frac{S_{N}}{\sigma_{N}}=\frac{NS_{1}}{\sqrt{N}\sigma_{1}}=\sqrt{N}{\mathrm{SNR}}_{1}$$

Equation (10) represents the ideal $\sqrt{N}$ SNR scaling predicted by the circuit-level noise model when independent white noise is dominant. This relationship should not be interpreted as a measured chip-level SNR result. The assumption that the noise components in different accumulation cycles are mutually uncorrelated is an idealized condition. In practical switched-capacitor readout circuits, 1/f noise, residual offset, switch charge injection, and clock feedthrough can introduce correlated or deterministic error components across repeated sensing cycles. These components are not fully averaged out by repeated accumulation and may be accumulated together with the capacitance-dependent signal component. In addition, practical fingerprint measurements can include measurement variations caused by finger contact pressure, skin condition, microscopic finger movement, and spatial non-uniformity. Therefore, the image-based ridge–valley SNR measured from fingerprint images may not follow this ideal $\sqrt{N}$ SNR scaling. For this reason, this work evaluates the effect of repeated charge accumulation in terms of both ridge–valley contrast enhancement and the stability of image-based ridge–valley SNR.

The ridge–valley contrast is used as the primary image-quality metric because it directly represents the separability of fingerprint ridge and valley patterns. It is defined as

(11) $$\mathrm{Contrast}=\frac{I_{\mathrm{valley}}-I_{\mathrm{ridge}}}{I_{\mathrm{valley}}+I_{\mathrm{ridge}}}$$

where *I*_ridge_ and *I*_valley_ are the average intensities of the ridge-candidate and valley-candidate regions, respectively. Contrast is reported as a percentage throughout this work.

The image-based ridge–valley SNR is used as an auxiliary image-quality metric to evaluate whether the measured fingerprint image remains stable as the accumulation number increases. In this work, it is defined as

(12) $$\mathrm{SNR}=\frac{\left|\mu_{\mathrm{valley}}-\mu_{\mathrm{ridge}}\right|}{\sqrt{\sigma_{\mathrm{valley}}^{2}+\sigma_{\mathrm{ridge}}^{2}}}$$

where *μ*_ridge_ and *μ*_valley_ are the mean intensities of the ridge-candidate and valley-candidate regions, respectively, and *σ*_ridge_ and *σ*_valley_ are the corresponding within-region population standard deviations. This metric reflects the mean separation and spatial dispersion of the ridge- and valley-candidate intensity distributions within the same region of interest. Therefore, it should not be interpreted as the physical SNR of the ADC, the input-referred noise SNR of the analog front-end, or the intrinsic noise floor of the chip circuit. The corresponding decibel value is calculated as SNR(dB) = 20 log_10_(SNR), where SNR is the linear ratio in Equation (12).

In this work, ridge–valley contrast is used as the main metric for evaluating fingerprint pattern separability, whereas the image-based ridge–valley SNR is used only to verify the relative stability of the measured fingerprint images. Because this SNR includes spatial variations caused by finger-contact conditions, skin conditions, pixel-to-pixel non-uniformity, and ridge/valley classification uncertainty, it is not expected to follow the ideal $\sqrt{N}$ circuit-level SNR scaling as the accumulation number *N* increases. A nearly constant image-based SNR does not, by itself, demonstrate that correlated temporal circuit noise dominates. If the mean ridge–valley separation and the within-region spatial standard deviations grow by a similar factor, their ratio can remain nearly unchanged without identifying the temporal noise correlations.

### 2.7. Chip Implementation and Measurement Setup

The proposed capacitive fingerprint sensor chip was fabricated using a 0.18 μm CMOS process. The sensor array consists of 144 × 40 capacitive sensing pixels with a pixel pitch of 50 μm, corresponding to a spatial resolution of approximately 508 dpi. The chip operates at a supply voltage of 3.3 V and integrates the column-shared charge-accumulation readout, an 8-bit ADC, timing control block, SPI, image buffer, and system control block. The main chip specifications are summarized in Table 1.

The accumulation-number-dependent capture time, current consumption, and frame energy are reported in the Results section.

**Figure 5:**
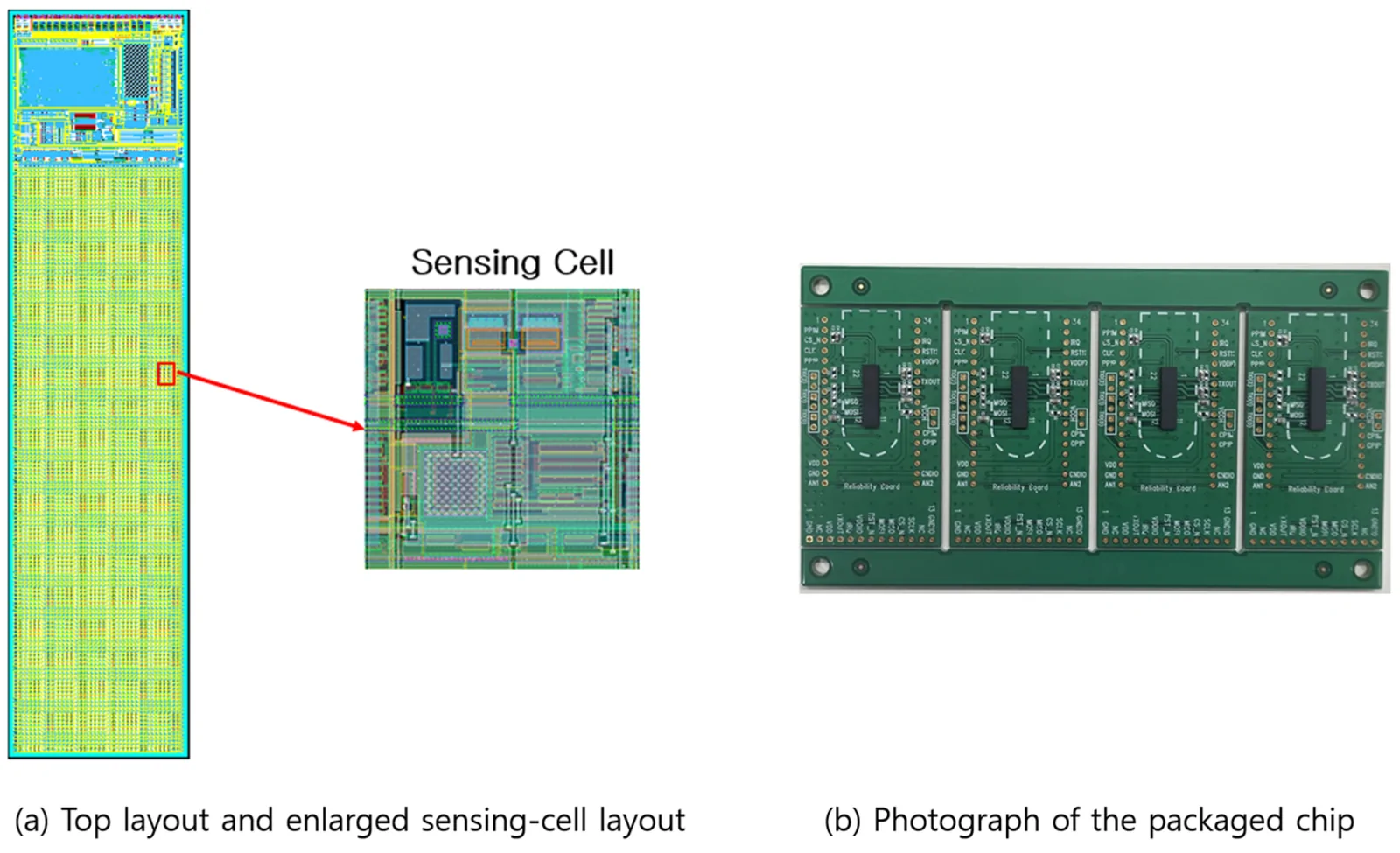
Fabricated capacitive fingerprint sensor chip.

The top layout, enlarged sensing-cell layout, and photograph of the packaged chip are shown in Figure 5. The fabricated chip includes the sensing array and peripheral readout/control circuits required for capacitive fingerprint image acquisition.

**Figure 6:**
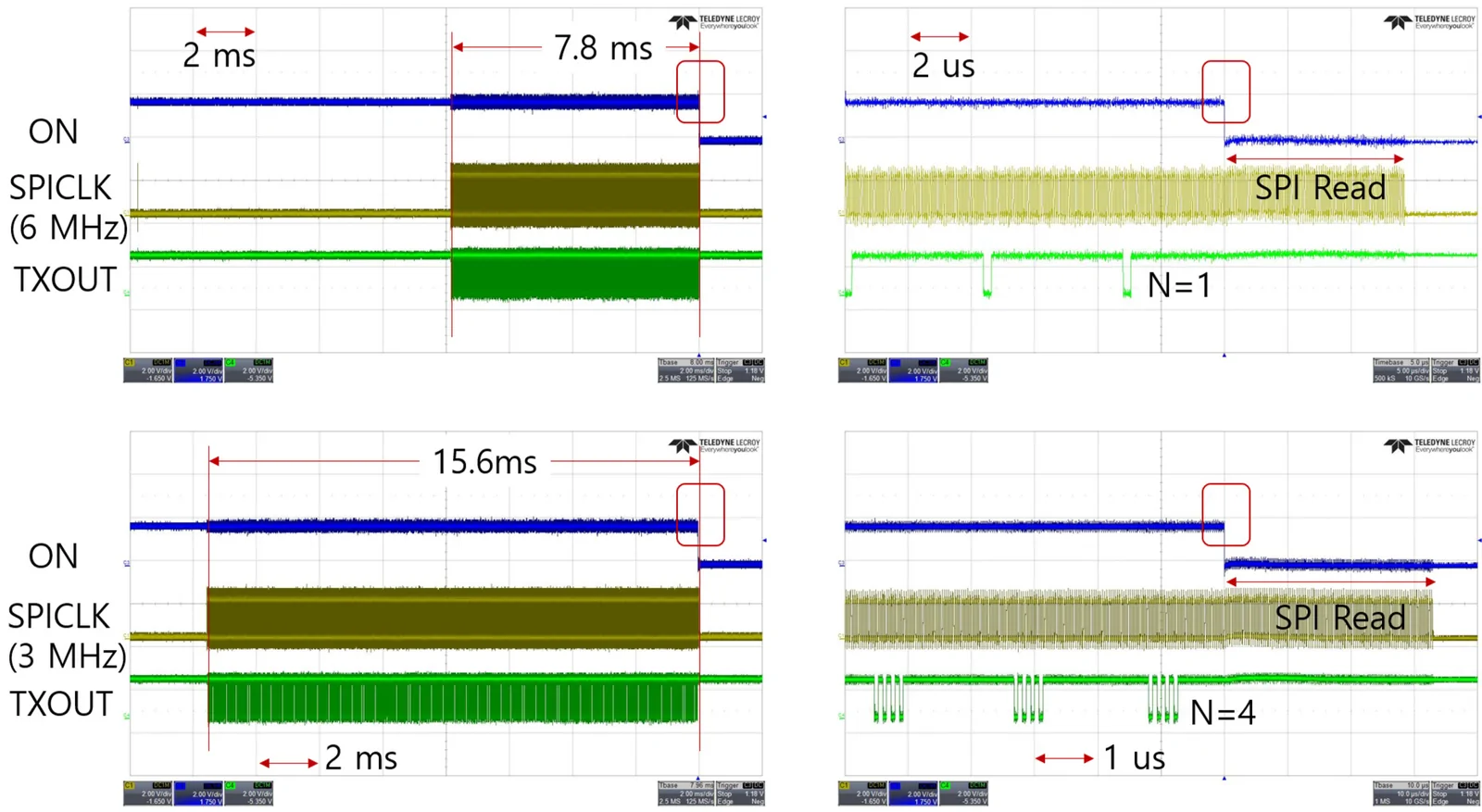
Representative operating waveforms of the fabricated chip measured under 3 MHz and 6 MHz SPI test conditions. Red double-headed arrows indicate the measured timing intervals.

Figure 6 shows representative operating waveforms of the fabricated chip measured under 3 MHz and 6 MHz SPI test conditions. The waveforms were measured using a WaveRunner 610Zi digital oscilloscope (LeCroy Corporation, Chestnut Ridge, NY, USA). The internal sensing-control clock TXOUT was brought out of the chip for external monitoring, and its measured waveform is included in Figure 6. These waveforms were used to verify the timing operation of the readout sequence and SPI data transfer under the measurement setup. The timing values shown in Figure 6 correspond to the waveform test conditions and are not intended to represent the optimized frame-capture times reported later in the Results section. Although the chip can operate at 3 MHz, this frequency was used for functional verification and was not included in the intended operating range for practical fingerprint acquisition.

The fabricated chip was evaluated using a packaged sensor board and external measurement equipment. All measurements were performed at room temperature, approximately 25 °C, with a supply voltage of 3.3 V. The package/passivation thickness under the measurement condition was 250 μm. Fingerprint images were acquired from two participants by ordinary finger contact without special pre-acquisition conditioning. Five acquisition sequences were performed for each participant. During each acquisition sequence, the participant’s finger was kept in contact with the sensor while the accumulation number was sequentially varied from *N* = 1 to *N* = 5, thereby reducing contact-position variation within the sequence. Thus, ten acquisition sequences were performed in total, resulting in ten fingerprint images for each accumulation number. The digitized output was mapped to an inverse gray scale, with analog outputs near the supply represented near gray level 0. Thus, the larger accumulated ridge output appears darker in the displayed image. The same gray-scale mapping and scaling gain were applied to all images at all accumulation settings, without per-image contrast normalization. Fingerprint-image analysis and quantitative calculations were performed using Microsoft Excel 2019 (MSO 16.0.10417.20208, 64-bit; Microsoft Corporation, Redmond, WA, USA) with Microsoft Visual Basic for Applications (VBA 7.1.1158).

All images were analyzed using the same region of interest (ROI) and the same ridge/valley classification criteria. The ROI was fixed to the same coordinate range for all measured images, defined as 4 ≤ *x* ≤ 135 and 3 ≤ *y* ≤ 36. Within the ROI, the lower 30% of the pixel-intensity distribution was classified as the ridge-candidate region, whereas the upper 30% was classified as the valley-candidate region. This image-based evaluation is consistent with the concept that fingerprint image quality is related to ridge strength, ridge continuity, ridge clarity, and ridge–valley structure integrity [8]. Pixel coordinates use 1-based indexing, with the upper-left pixel defined as (1,1), *x* increasing to the right, and *y* increasing downward. The ROI contains 4488 pixels (132 × 34). Pixel intensities were sorted automatically, and the lowest 1346 and highest 1346 values (⌊0.30 × 4488⌋) were selected for the ridge- and valley-candidate groups, respectively; the remaining 1796 values were excluded. These are intensity-ranked candidate groups, not manually traced or independently ground-truth-labeled regions.

The upper and lower 30% intensity regions were selected as a practical compromise between ridge–valley selectivity and the stability of the image-domain statistics. A smaller percentile threshold emphasizes the most extreme ridge and valley intensities and therefore tends to increase the measured contrast, but it uses fewer pixels and can be more sensitive to local variations, isolated extreme pixel values, and spatial nonuniformity.

In contrast, a larger percentile threshold includes more pixels but increasingly incorporates intermediate-intensity pixels near ridge–valley transition regions, thereby reducing selectivity. Thus, the 30% threshold was not selected to maximize the reported contrast or image-based ridge–valley SNR, but to provide a sufficiently large pixel population while excluding the central-intensity transition region.

For each accumulation number, one frame with the lowest image-based ridge–valley SNR under the 30% criterion was excluded according to the image-quality screening rule, and the remaining nine images were retained for calculation of the averaged values. This screening procedure was used for image-quality evaluation and was not based on an independent statistical outlier test. The possible causes of the low-SNR frames are discussed in Section 3.3.

To examine the sensitivity of the results to the percentile selection criterion, the same nine retained frames were additionally analyzed using lower and upper 10%, 20%, 30%, and 40% intensity regions. The same ROI and percentile-based classification rule were applied for all accumulation numbers so that the effect of *N* could be compared under consistent analysis conditions. For each *N*, the same nine frames retained under the 30% screening criterion were used for all four percentile criteria. The reported average SNR for each percentile criterion was calculated as the arithmetic mean of the per-image SNR values in dB.

## 3. Results

This section presents the measured fingerprint images and evaluates the effect of the accumulation number *N* on ridge–valley contrast, image-based ridge–valley SNR, capture time, and energy consumption.

### 3.1. Representative Fingerprint Images

The representative images for *N* = 1–5 used in Table 2 and the fixed-coordinate analysis were obtained from the same acquisition sequence. The representative acquisition sequence was selected based on the ridge–valley contrast and image-based ridge–valley SNR calculated using the same region of interest and ridge/valley classification criteria. At *N* = 1, the brightness difference between ridge and valley regions is relatively small, and the fingerprint pattern exhibits low contrast. As the accumulation number increases, the ridge–valley separability gradually improves. At *N* = 5, the ridge pattern becomes more clearly visible, indicating that the proposed charge-accumulation readout effectively enhances the ridge–valley signal separation caused by the small capacitance difference.

Based on the representative images, the ridge–valley contrast increased from 6.46% at *N* = 1 to 45.58% at *N* = 5, corresponding to a contrast ratio of approximately 7.06. The image-based ridge–valley SNR remained in the range of 13.86–14.28 dB. For this representative sequence, the ridge–valley contrast increased substantially, while the image-based ridge–valley SNR remained relatively stable.

### 3.2. Statistical Evaluation of Repeated Measurements

To evaluate the image-quality improvement more reliably, ten fingerprint images were acquired for each accumulation number, consisting of five acquisition sequences from each of the two participants. All images were analyzed using the same ROI and the same ridge/valley classification criteria. Table 3 reports mean ± sample standard deviation for the two image-quality metrics calculated from the nine retained frames per accumulation number, as described in Section 2.7. The timing, current, and energy results are also reported for the corresponding SPI/readout settings.

To quantify the trade-off between ridge–valley contrast and frame-energy consumption, an Energy-Contrast Efficiency (ECE) metric is also introduced. In this work, ECE is defined as ECE(*N*) = *C*_avg_(*N*)/*E*_frame_(*N*), where *C*_avg_(*N*) is the average ridge–valley contrast, expressed as a percentage, at accumulation number *N*, and *E*_frame_(*N*) is the frame energy estimated using the supply voltage, frame-averaged operation current, and capture time, as described in Section 3.5. The unit of ECE is %/μJ. A higher ECE indicates that a larger ridge–valley contrast is obtained per unit frame energy.

The average ridge–valley contrast increased from 5.71% at *N* = 1 to 12.54% at *N* = 2, 21.09% at *N* = 3, 31.76% at *N* = 4, and 43.86% at *N* = 5. Therefore, the average ridge–valley contrast increased by a factor of approximately 7.68 from *N* = 1 to *N* = 5. In contrast, the average image-based ridge–valley SNR varied only slightly with *N* and remained above 12 dB for all accumulation numbers. The ECE increased from 0.223%/μJ at *N* = 1 to 0.525%/μJ at *N* = 5, indicating that the ridge–valley contrast increased proportionally more than the frame energy within the tested range. However, the capture time and frame energy also increased with *N*. Therefore, the accumulation number should be selected by considering the required ridge–valley contrast, energy efficiency, capture time, and power budget of the target application. These results indicate that the proposed repeated charge-accumulation readout enhances ridge–valley signal separability and image contrast while the image-based ridge–valley SNR remains relatively stable.

The sample standard deviations in Table 3 were calculated across the nine retained frame-level metric values using the *n* − 1 denominator (*n* = 9). They describe between-frame variability and are distinct from the within-image spatial intensity standard deviations used in Equation (12). These frame-level statistics pool the retained acquisitions from the two participants and should not be interpreted as participant-level variability. These descriptive statistics characterize the retained acquisitions only; the exclusion of the lowest-SNR frame can favor higher-SNR observations, and the present dataset does not establish population-level repeatability or statistical significance.

As a sensitivity check, the metrics were also summarized using all ten frames for each accumulation number. The mean contrast still increased monotonically from 5.58% at *N* = 1 to 43.73% at *N* = 5, while the mean image-based ridge–valley SNR ranged from 11.68 to 11.97 dB; therefore, the statement that the mean SNR remained above 12 dB applies specifically to the nine-frame results after screening.

To further evaluate the sensitivity of the image-quality results to the percentile selection criterion, a threshold-sensitivity analysis was performed using lower and upper 10%, 20%, 30%, and 40% intensity regions, as summarized in Table 4.

The threshold-sensitivity analysis showed that the absolute contrast and image-based ridge–valley SNR values depended on the selected percentile, as expected. Smaller percentile thresholds resulted in larger absolute contrast values because they selected more extreme ridge and valley intensity populations. A similar threshold dependence was observed for the image-based ridge–valley SNR. For the 10%, 20%, 30%, and 40% criteria, the corresponding average image-based ridge–valley SNR values over *N* = 1–5 ranged from 18.85 to 19.59 dB, 15.32 to 15.76 dB, 12.31 to 12.65 dB, and 9.44 to 9.76 dB, respectively. However, the overall dependence on the accumulation number *N* remained consistent over the tested threshold range. From *N* = 1 to *N* = 5, the average ridge–valley contrast increased from 7.91% to 58.01%, from 6.68% to 50.34%, from 5.71% to 43.86%, and from 4.85% to 37.76% for the 10%, 20%, 30%, and 40% thresholds, respectively. These values correspond to contrast ratios of approximately 7.33×, 7.54×, 7.68×, and 7.79×, respectively.

**Figure 7:**
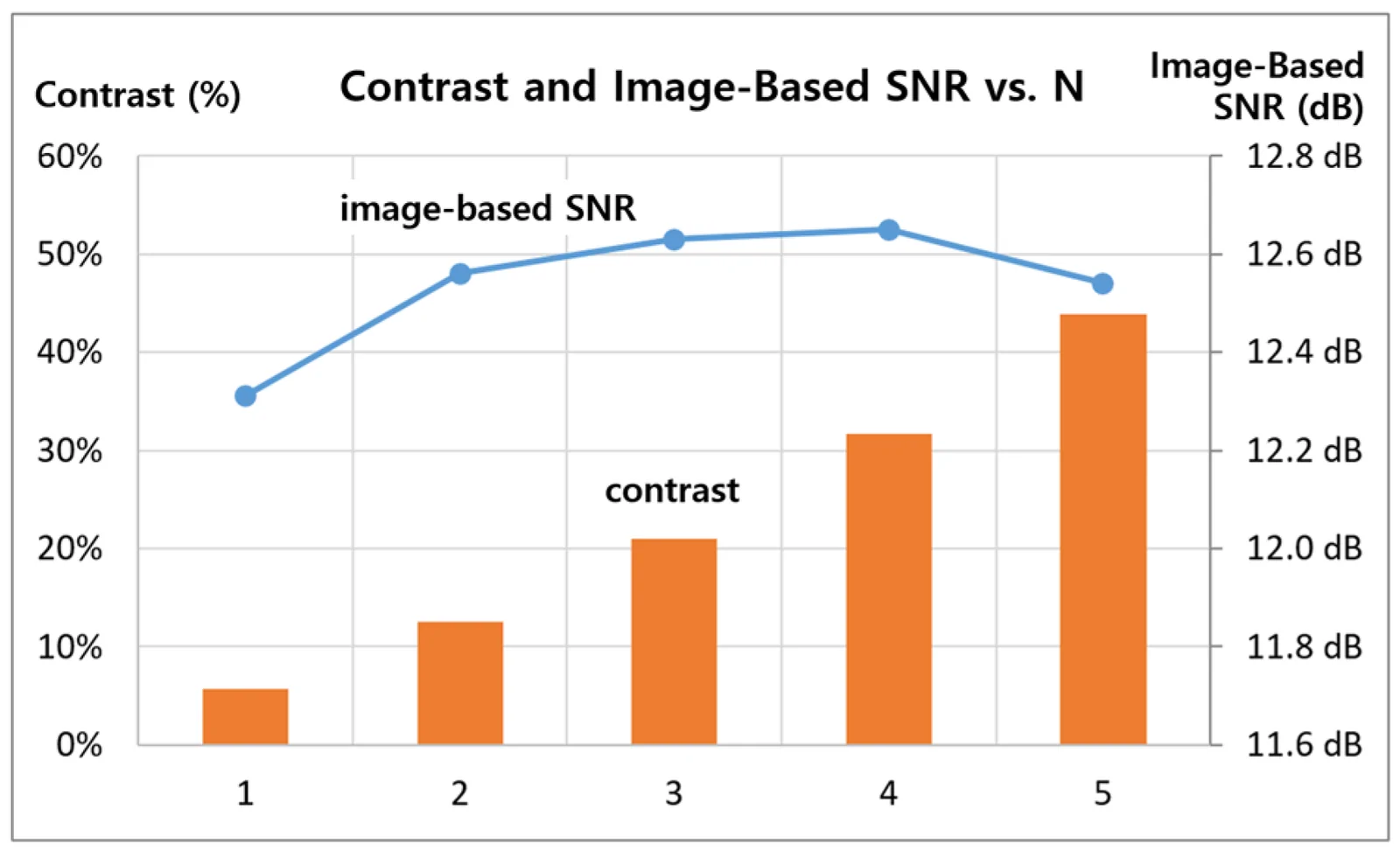
Average ridge–valley contrast and image-based ridge–valley SNR as a function of accumulation number *N*. The plotted values are means over the nine retained frames per accumulation number; the corresponding sample standard deviations are reported in Table 3.

The image-based ridge–valley SNR also remained relatively stable with increasing *N* for each fixed threshold, although its absolute value decreased as the selected percentile range became wider. These results indicate that the observed contrast enhancement with increasing *N* is not specific to the 30% threshold and that the main trend remains consistent over the tested 10–40% percentile range. The limited variation in image-based ridge–valley SNR can be understood from the definition of the SNR used in this work. Unlike the ideal $\sqrt{N}$ SNR scaling in Equation (10), which is based on an ideal circuit-level model with uncorrelated per-cycle noise, the image-based SNR in Equation (12) is calculated from the spatial intensity distributions of ridge-candidate and valley-candidate pixels in each measured fingerprint image. Therefore, these image-based SNR values should not be interpreted as the physical ADC SNR, input-referred front-end SNR, or circuit noise floor of the chip. These results indicate that the proposed charge-accumulation readout substantially increases ridge–valley contrast while the image-based ridge–valley SNR remains relatively stable under the measured image-acquisition conditions. Figure 7 summarizes the average contrast and image-based ridge–valley SNR as functions of the accumulation number.

**Figure 8:**
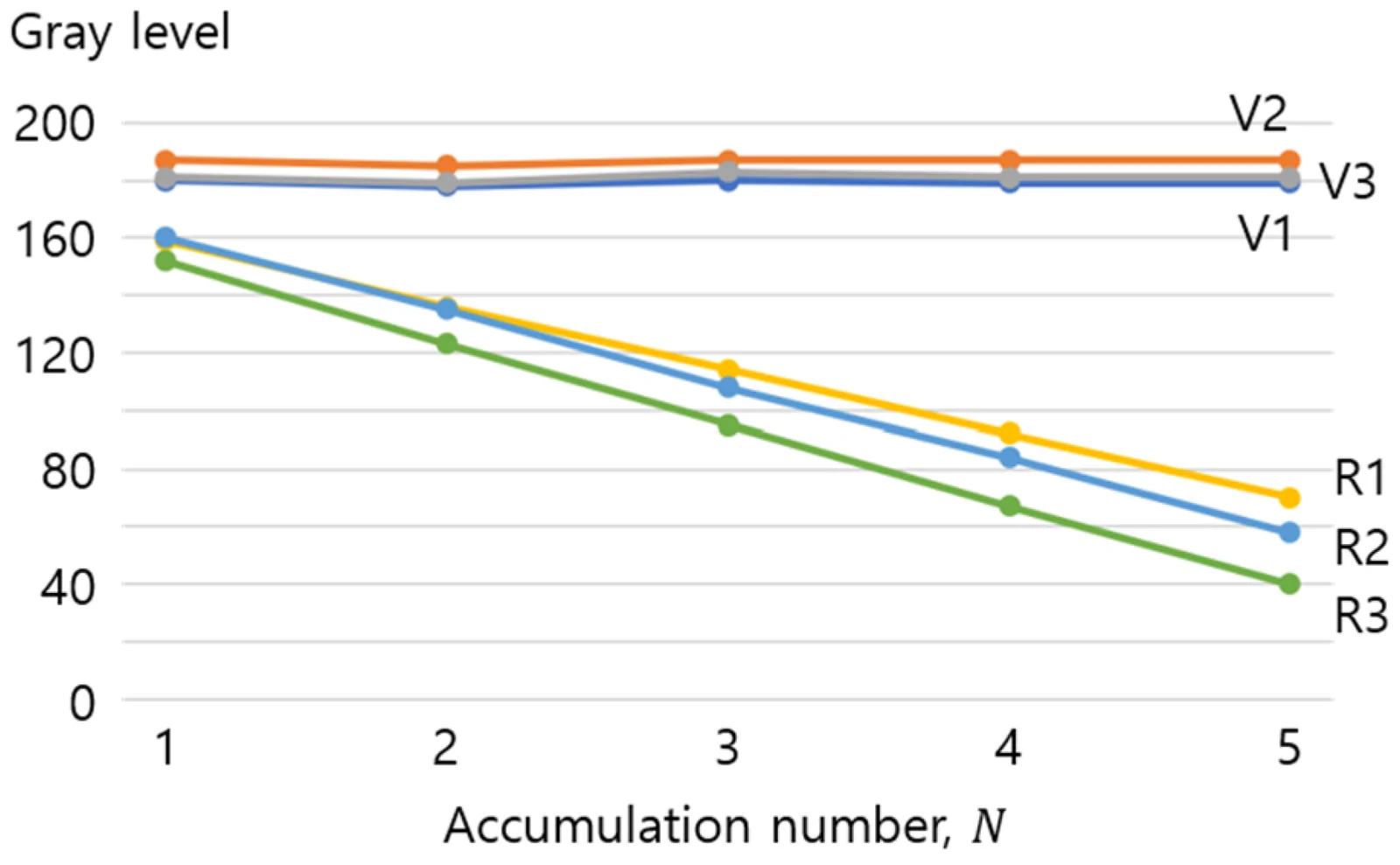
Gray-level variations at three fixed ridge pixels (R1–R3) and three fixed valley pixels (V1–V3) as a function of accumulation number *N*.

To further examine the accumulation effect independently of the ROI-averaged analysis, gray-level variations were evaluated at representative fixed sensor pixel coordinates located within visually consistent ridge and valley regions. Three ridge coordinates and three valley coordinates were selected, and the gray levels at the same sensor pixel coordinates were evaluated across the representative images acquired at *N* = 1–5. The corresponding pixel coordinates and gray-level values are summarized in Table 5, and the individual fixed-pixel trajectories are shown in Figure 8.

The average gray level of the three selected valley pixels remained nearly constant, varying from 182.67 at *N* = 1 to 182.33 at *N* = 5. In contrast, the average gray level of the three selected ridge pixels progressively decreased from 157.00 to 56.00 as *N* increased from 1 to 5. Consequently, the average ridge–valley gray-level separation increased from 25.67 at *N* = 1 to 126.33 at *N* = 5, corresponding to a separation ratio of approximately 4.92. This fixed-coordinate analysis provides complementary evidence that repeated charge accumulation progressively enhances the local gray-level separation between representative ridge and valley regions.

Although finger contact with the sensor was maintained throughout the representative *N* = 1–5 acquisition sequence, exact anatomical registration across these images was not independently verified, and small variations due to microscopic finger motion or contact nonuniformity may remain. Therefore, the fixed-pixel analysis is intended as a complementary readout-level verification rather than as exact tracking of an identical anatomical point on the finger.

### 3.3. Screened-Frame Analysis

For each accumulation condition, some frames exhibited substantially lower image-based ridge–valley SNR than the other frames obtained under the same condition. These frames may be associated with measurement-related variations, including nonuniform finger contact, local pressure variation, skin-condition variation, microscopic movement, and spatial nonuniformity [2,6,8]. Their causes were not independently identified in this study.

Previous studies have reported that capacitive fingerprint image quality can be degraded by finger-surface conditions, such as wet or dry fingers, as well as by noisy environments [2]. Increased finger-surface resistance under dry-finger conditions can also degrade ridge–valley contrast and image quality [6]. In addition, fingerprint acquisition can be affected by contact pressure, dryness/wetness, dirt, noise, and residual prints, which may produce weak impressions or nonuniform contrast [8].

Therefore, all images were analyzed using the same ROI and the same ridge/valley classification criteria, and the averaged values were calculated after excluding the lowest-SNR frame for each accumulation number, as described in Section 2.7. This procedure was used to evaluate the overall trend of image-quality improvement based on repeated measurements rather than relying on a single frame.

To further discuss the expected behavior of the proposed readout under practical acquisition conditions, the effects of dry/wet fingers, pressing force, and media thickness can be considered from the capacitive sensing principle. A dry finger, insufficient pressing force, or increased media/passivation thickness increases the effective sensing distance or reduces the effective contact area, thereby reducing the ridge–valley capacitance difference and the single-cycle readout signal. Under such reduced-signal conditions, repeated charge accumulation is expected to be beneficial because it accumulates the capacitance-dependent charge-domain signal over multiple sensing cycles, as long as the accumulated output remains within the allowable dynamic range.

In contrast, excessive moisture, excessive pressing force, severe contact nonuniformity, residual prints, and local contamination can introduce nonuniform ridge deformation, parasitic coupling, or local saturation, which cannot be fully compensated by charge accumulation alone. Therefore, the proposed charge-accumulation readout is expected to improve ridge–valley contrast under reduced-signal conditions, such as weak contact or increased sensing distance, but its robustness under controlled dry/wet finger conditions, pressing-force variations, and media-thickness variations should be further verified through controlled experiments using a larger dataset.

### 3.4. Relationship to Fingerprint Recognition Performance

This work does not claim a direct improvement in recognition rate, false acceptance rate (FAR), or false rejection rate (FRR) based on a fingerprint matching algorithm. Instead, the proposed readout was evaluated in terms of ridge–valley contrast and image-based ridge–valley SNR to determine whether it provides recognition-relevant image-quality improvement. Previous studies have reported that fingerprint image quality is related to ridge strength, ridge continuity, ridge clarity, and ridge–valley structure integrity, and that such quality factors are closely associated with minutiae-based and ridge-based verification performance [8]. In automatic fingerprint matching, minutiae extraction is a key step, and poor image quality can cause spurious minutiae, missing genuine minutiae, and localization errors. Therefore, improved ridge–valley clarity can provide more favorable image data for subsequent minutiae extraction and matching processes [15].

The SNR used in this work is not the ADC quantization-noise SNR or the input-referred circuit-noise SNR. Rather, it is an image-based ridge–valley SNR calculated by normalizing the mean intensity difference between ridge-candidate and valley-candidate regions by the intensity variations within the two regions. Therefore, this metric includes not only circuit noise but also finger-contact condition, skin condition, pixel-to-pixel non-uniformity, spatial variation in the fingerprint pattern, and ridge/valley candidate-selection variation. Accordingly, the absolute value of this SNR should not be converted to or interpreted as ADC ENOB. Instead, it is used as an image-based metric for evaluating the relative change and stability of ridge–valley separability with respect to the accumulation number *N*.

In capacitive fingerprint sensors, the signal difference between ridge and valley regions should be sufficiently preserved in the digital output after ADC conversion. Previous work has reported that, under fixed analog front-end and ADC conditions, the average capacitance difference Δ*C*_avg_ can be used as a metric for evaluating sensing-part performance, and that the implemented fingerprint matching algorithm may require an ADC ENOB of 3 bits for the Δ*C*_avg_ signal [13]. The application-specific 3-bit ENOB requirement reported in [13] provides a useful design reference for interpreting the digitization of weak ridge–valley signals. It is not presented as an experimentally verified ENOB of the present chip. Therefore, the ridge–valley contrast enhancement observed in this work can be interpreted as an increase in the effective digital separability between ridge and valley patterns, which may provide more favorable image data for subsequent fingerprint matching algorithms.

In the nine-frame results after screening, the average image-based ridge–valley SNR remained above 12 dB for all accumulation numbers, while the average ridge–valley contrast increased by a factor of approximately 7.68 from *N* = 1 to *N* = 5. Although these results do not demonstrate direct improvements in FAR or FRR, they indicate that the proposed repeated charge-accumulation readout substantially improves fingerprint pattern visibility and ridge–valley separability. Established fingerprint image-quality measures evaluate multiple characteristics, including local ridge–valley clarity, orientation certainty, and ridge-flow continuity [8]. In this work, ridge–valley contrast is used as the primary metric because it directly quantifies the intensity separation targeted by the proposed charge-accumulation readout. This separation is an important aspect of fingerprint image quality, but contrast alone does not establish overall image quality or matching performance. A quantitative comparison with applicable standardized fingerprint-quality metrics is left for future work.

### 3.5. Power and Capture-Time Trade-Off

Increasing the accumulation number *N* requires additional sensing and accumulation cycles for the same pixel, thereby increasing the frame capture time and frame energy [9,11]. As shown in Table 3, the capture time increased from 6.360 ms at *N* = 1 to 11.358 ms at *N* = 5, and the frame-averaged operation current increased from 1.22 mA to 2.23 mA. The active sensing current also increased from 6.78 mA to 8.49 mA over the tested operating settings, consistent with the increased sensing activity required for repeated accumulation.

In terms of area, increasing the accumulation number *N* does not increase the pixel-level circuit area after fabrication because *N* is an operation parameter. The proposed architecture performs charge accumulation using the column-shared accumulation integrator rather than adding an accumulation circuit inside each pixel. Therefore, the accumulation-related area overhead is associated with the column-shared readout circuit, including the accumulation capacitor and switches, while the power and capture-time overhead increase with *N*.

The frame energy was estimated using

*E*_frame_ = *V*_DD_*I*_avg_*T*_capture_ (13)

where *V*_DD_ is the supply voltage, *I*_avg_ is the frame-averaged operation current, and *T*_capture_ is the frame capture time. The chip operates synchronously with the SPI clock, and the acquisitions summarized in Table 3 used SPI/readout clock settings of 4–8 MHz, with 4 MHz used at *N* = 5. SPI frequencies below 4 MHz were not included in the intended operating range for practical fingerprint acquisition. Active sensing current refers to the sensing interval; frame-averaged operation current includes the chip’s normal operation, including SPI transfer, during acquisition. The timing and energy results therefore characterize these operating settings rather than a sweep at one fixed SPI frequency.

For *V*_DD_ = 3.3 V, the estimated frame energy increased from approximately 25.6 μJ at *N* = 1 to 83.6 μJ at *N* = 5. From an energy–contrast trade-off perspective, *N* = 5 provides the highest measured ridge–valley contrast and the highest ECE in this work, but it also requires the largest frame energy and capture time. Therefore, a smaller accumulation number may be selected when lower latency or lower frame energy is more important than maximum contrast enhancement. In practical applications, the accumulation number should be selected by considering the required image contrast, frame rate, frame energy, active sensing current, dynamic range, and energy efficiency.

### 3.6. Comparison with Previous Works

Previous CMOS capacitive fingerprint sensor studies have improved ridge–valley capacitance detection and fingerprint image quality using differential sensing, charge-sharing detection, charge-transfer sensing, feedback capacitive sensing, low-noise analog front-ends, and image-quality improvement techniques [2,3,4,5,6,7]. Other studies have reported single-chip fingerprint sensors or micromachined capacitive sensor arrays [16,17]. In the broader field of capacitive sensor interfaces, switched-capacitor charge-domain readout, autozeroing/CDS techniques, and charge-overflow reduction or sensing-range extension techniques have also been reported [10,11,12].

Previous studies have addressed image quality and sensing sensitivity through pixel-level adaptive image enhancement [18], charge-sharing detection with parasitic-capacitance compensation [19], differential integration [2], finger-surface potential control [6], and micromachined capacitive sensing [17]. Transparent half-diamond mutual-capacitive sensors [20] and sensing-mode reconfigurable analog front-ends for capacitive touch and a-IGZO TFT-based active-matrix fingerprint sensors [21] have also been reported. For under-glass fingerprint sensing, differential coded multiple signaling with a fully differential receiver [22] and differential phase-encoded driving with an on-chip replica channel [7] have been employed.

Against this background, the present work applies a column-shared charge-accumulation integrator to the column readout path of a CMOS capacitive fingerprint sensor. The proposed readout repeatedly senses the same pixel and accumulates the resulting charge-domain signals before ADC conversion, thereby enhancing ridge–valley contrast without increasing pixel-level circuit complexity. The fabricated 0.18 μm CMOS chip was experimentally evaluated using actual fingerprint images acquired for accumulation numbers from *N* = 1 to *N* = 5. The average ridge–valley contrast increased from 5.71% at *N* = 1 to 43.86% at *N* = 5, corresponding to a contrast ratio of approximately 7.68, while the mean image-based ridge–valley SNR remained above 12 dB in the nine-frame results after screening. Capture time and current consumption were also measured, and frame energy was calculated from these measurements.

Table 6 provides a quantitative comparison with representative CMOS capacitive fingerprint sensors and readout circuits in terms of CMOS process, array and pixel dimensions, spatial resolution, frame rate, power consumption, and reported contrast-, SNR-, or sensitivity-related metrics. Because the previous studies employ different sensor structures, sensing conditions, readout architectures, operating modes, and performance metrics, the reported metrics are not directly comparable numerically. Parameters that were not available in the corresponding publications are denoted as N/R (not reported), and the reported contrast-, SNR-, and sensitivity-related metrics are presented using the definitions adopted in the respective publications.

## 4. Discussion

The measurement results demonstrate that the proposed repeated charge-accumulation readout is effective for enhancing ridge–valley contrast in a CMOS capacitive fingerprint sensor. As the accumulation number *N* increases, the small charge-domain signals generated from the same pixel are repeatedly accumulated by the column-shared charge-accumulation integrator. Consequently, the average intensity difference between ridge and valley regions increases, resulting in improved ridge–valley contrast [11].

After applying the image-quality screening rule, the average ridge–valley contrast increased from 5.71% at *N* = 1 to 43.86% at *N* = 5, corresponding to a contrast ratio of approximately 7.68. In contrast, the average image-based ridge–valley SNR remained above 12 dB for all accumulation numbers. These image-based results do not establish whether the ADC SNR or the intrinsic circuit SNR changes with the accumulation number. The proposed readout substantially enhances ridge–valley signal separability and image contrast, while the image-based ridge–valley SNR remains relatively stable.

However, increasing the accumulation number *N* introduces trade-offs among capture time, frame energy, current consumption, and dynamic range. In the measured results, the capture time increased from 6.360 ms at *N* = 1 to 11.358 ms at *N* = 5, while the estimated frame energy increased from 25.6 μJ to 83.6 μJ. The active sensing current also increased from 6.78 mA to 8.49 mA over the tested operating settings, consistent with the increased sensing activity required for repeated accumulation. In addition, excessive accumulation may cause the accumulated output to exceed the sensing range of the readout circuit, the output swing, or the ADC input range [12]. Therefore, the accumulation number should be selected by considering the required ridge–valley contrast, capture time, frame energy, current consumption, and dynamic range.

The physical noise floor of the ADC and analog front-end was not independently measured as a function of the accumulation number *N* in this work. Such circuit-level characterization would require a separate measurement setup using a fixed input capacitance or a calibrated capacitance source under controlled-input conditions. Therefore, this work does not claim an improvement in ADC SNR, input-referred front-end noise floor, or intrinsic circuit noise floor. The reported image-based ridge–valley SNR is used only as an image-domain metric to evaluate the stability of ridge–valley separability in the acquired fingerprint images. In addition, the cycle-to-cycle behavior and correlation of circuit-level noise and error components, including 1/f noise, residual DC offset, switch charge injection, and clock feedthrough, were not independently measured in this work. Therefore, the proposed readout does not claim to eliminate accumulated DC bias or low-frequency noise completely. A detailed circuit-level noise characterization using a fixed or calibrated capacitance source remains as future work.

Fixed-pattern noise (FPN) refers to reproducible pixel- or column-dependent response differences. A fixed per-cycle offset is not averaged out by repeated sensing of the same pixel and can accumulate with the signal. The spatial dispersion in Equation (12) can include FPN, but also includes genuine fingerprint structure and contact variations; it therefore cannot isolate FPN. No quantitative FPN reduction is claimed here. Uniform-input measurements and repeated response maps are needed to separate fixed response variations from temporal noise.

Practical fingerprint measurements can also be affected by nonideal acquisition conditions, including finger contact pressure, skin moisture, contamination, residual prints, and contact nonuniformity [2,6,8]. Structural factors such as package and passivation thickness can also affect the ridge–valley capacitance difference and fingerprint image quality [13]. Therefore, although the proposed readout improves ridge–valley contrast under the measured conditions, its performance should be further evaluated under diverse finger and packaging conditions. Accordingly, the robustness discussion in this work should be interpreted as a readout-level trend expected under reduced capacitance-difference conditions, rather than as complete experimental verification under all practical finger and packaging conditions.

This work does not claim a direct improvement in fingerprint recognition accuracy, FAR, or FRR. Instead, it demonstrates recognition-relevant image-quality improvement by enhancing ridge–valley contrast while the image-based ridge–valley SNR remains relatively stable. Future work will include evaluation with a larger number of users and fingers and quantitative recognition-level metrics such as FAR, FRR, and minutiae-extraction success rate.

## 5. Conclusions

This paper presented a CMOS capacitive fingerprint sensor chip with a column-shared charge-accumulation readout for ridge–valley contrast enhancement. The proposed readout repeatedly senses the same pixel and accumulates the resulting charge-domain signals at the column level before analog-to-digital conversion. This approach enhances ridge–valley signal separability without increasing pixel-level circuit complexity.

The chip was fabricated using a 0.18 μm CMOS process and includes a 144 × 40 capacitive sensor array with a 50 μm pixel pitch, corresponding to approximately 508 dpi. Measurements covered the full implemented accumulation range from *N* = 1 to *N* = 5. After applying the screening rule described in Section 2.7, the mean ridge–valley contrast across the nine retained frames increased from 5.71 ± 0.72% at *N* = 1 to 43.86 ± 4.97% at *N* = 5, corresponding to a contrast ratio of approximately 7.68. The reported ± values denote between-frame sample standard deviations. The mean image-based ridge–valley SNR remained within 12.31–12.65 dB across these retained-frame datasets. The all-ten-frame sensitivity analysis also preserved the increasing contrast trend, although the mean image-based ridge–valley SNR values were lower.

The trade-off analysis shows that ridge–valley contrast and Energy-Contrast Efficiency increased with *N* within the tested range, while capture time and frame energy also increased. Therefore, the accumulation number should be selected according to the required contrast, frame rate, energy budget, and dynamic-range constraints of the target application.

The demonstrated benefit is readout-level contrast enhancement under the reported acquisition conditions, rather than a verified improvement in intrinsic circuit noise or fingerprint matching accuracy. Quantitative circuit-noise and FPN characterization, the experimental saturation boundary, and standardized fingerprint-quality benchmarking remain to be established. Future work will include controlled-input noise and FPN measurements, saturation characterization using an implementation with an extended accumulation range, and evaluation with larger fingerprint datasets under varied finger and contact conditions. The latter will include applicable standardized image-quality metrics and recognition-level measures such as FAR, FRR, and minutiae-extraction success rate.

## Figures and Tables

**Table 1:** Basic specifications of the fabricated capacitive fingerprint sensor chip.

Item	Value	Comment
Process	180 nm CMOS	1-poly, 3-metal
Supply voltage	3.3 V	-
Pixel pitch/resolution	50 μm/508 dpi	-
Sensor array	144 × 40	capacitive sensor array
Chip size	9.24 mm × 2.25 mm	with scribe line, 80 μm × 80 μm
Nominal clock frequency	20 MHz	internal oscillator
Standby current	<1 μA	standby mode
Finger detection time	<1.0 ms	at 10 finger-detection zones
Package/passivation thickness	250 μm	-

**Table 2:** Ridge–valley contrast and image-based SNR of representative fingerprint images.

Accumulation Number	Representative Fingerprint Image	Contrast (%)	Image-Based SNR (Linear)	Image-Based SNR (dB)
*N* = 1	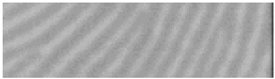	6.46%	4.93	13.86 dB
*N* = 2	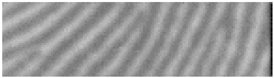	14.13%	5.02	14.01 dB
*N* = 3	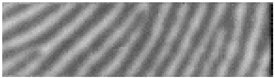	23.39%	5.14	14.23 dB
*N* = 4	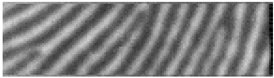	34.38%	5.11	14.17 dB
*N* = 5	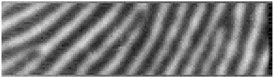	45.58%	5.18	14.28 dB

**Table 3:** Repeated-measurement results of ridge–valley contrast, image-based SNR, capture time, operation current, frame energy, active sensing current, and Energy-Contrast Efficiency (ECE).

Accumulation Number	Average Contrast (%)	Image-Based SNR (dB)	Capture Time (ms)	Average Operation Current (mA)	Frame Energy (μJ)	ECE (%/μJ)	Active Sensing Current (mA)
*N* = 1	5.71 ± 0.72	12.31 ± 1.19	6.360	1.22	25.6	0.223	6.78
*N* = 2	12.54 ± 1.74	12.56 ± 1.34	7.378	1.53	37.3	0.336	7.35
*N* = 3	21.09 ± 2.84	12.63 ± 1.35	8.384	1.78	49.2	0.429	7.94
*N* = 4	31.76 ± 4.02	12.65 ± 1.43	9.706	2.01	64.4	0.493	8.24
*N* = 5	43.86 ± 4.97	12.54 ± 1.48	11.358	2.23	83.6	0.525	8.49

Note: For each accumulation number, ten fingerprint images were acquired from two participants (five acquisition sequences per participant), and one frame with the lowest image-based ridge–valley SNR under the 30% criterion was excluded according to the image-quality screening rule. Contrast and image-based ridge–valley SNR in Table 3 are reported as mean ± sample standard deviation across the remaining nine images (*n* = 9). Frame energy was estimated from the supply voltage, frame-averaged operation current, and capture time, as described in Section 3.5, and ECE was calculated using the definition given above.

**Table 4:** Threshold-sensitivity analysis of average ridge–valley contrast using different percentile criteria.

Threshold	*N* = 1	*N* = 2	*N* = 3	*N* = 4	*N* = 5	Contrast Ratio (*N* = 5/*N* = 1)
10%	7.91%	17.12%	28.51%	42.49%	58.01%	7.33×
20%	6.68%	14.57%	24.39%	36.56%	50.34%	7.54×
30%	5.71%	12.54%	21.09%	31.76%	43.86%	7.68×
40%	4.85%	10.71%	18.05%	27.32%	37.76%	7.79×

**Table 5:** Gray-level variations at selected fixed ridge and valley sensor pixel coordinates for different accumulation numbers.

Region	Pixel	Coordinate	*N* = 1	*N* = 2	*N* = 3	*N* = 4	*N* = 5
Valley	V1	(30, 8)	180	178	180	179	179
Valley	V2	(99, 22)	187	185	187	187	187
Valley	V3	(58, 36)	181	179	183	181	181
Ridge	R1	(60, 13)	159	136	114	92	70
Ridge	R2	(106, 15)	160	135	108	84	58
Ridge	R3	(27, 31)	152	123	95	67	40

**Table 6:** Quantitative comparison with representative CMOS capacitive fingerprint sensors and readout circuits.

Ref. Year/Venue	Process	Array/Pixel Size or Pitch/Resolution	Frame Rate (fps)	Power (mW)	Reported Metric
[18] 2002/ISSCC	0.6 μm CMOS	210 × 100 50 × 50 μm^2^ 500 dpi	N/R	40	Quantitative contrast/SNR gain: N/R.
[19] 2005/JSSC	0.35 μm CMOS	160 × 192 60 μm pitch 423 dpi	N/R	35 mW (sensing + identification)	Ridge–valley ΔV: 0.61 → 1.72 V; simulation.
[6] 2010/JSSC	0.5 μm CMOS + sensor process	224 × 256 50 × 50 μm^2^ 508 dpi	0.3–50 fps	25	Quantitative contrast/SNR gain: N/R.
[17] 2012/IEEE Sensors J	0.35 μm CMOS + micromachining	8 × 32 65 × 65 μm^2^ 390 dpi	N/R	N/R	Sensitivity: 1.14 fF/MPa; simulation ^a^
[20] 2016/IEEE Sensors J.	0.18 μm CMOS readout; glass sensor	72 × 72 75 μm Electrode pitch 322 CPI	N/R	N/R	Touch/no-touch ΔC: 210 fF; 2.3× versus linear pattern.
[2] 2018/Sensors	0.13 μm CMOS	20 × 16 90 × 90 μm^2^ cell 282.2 dpi ^b^	5000 ^c^	N/R	Simulated SNR gain: 53.37–53.39 dB ^c^.
[21] 2019/IEEE Sensors J.	0.13 μm CMOS AFE; a-IGZO TFT sensor	96 × 96 50.8 × 50.8 μm^2^ 500 ppi	3	65.31 (AFE IC)	Fingerprint SNR: 25.0 dB; 0.15 mm cover glass.
[22] 2020/IEEE TCAS-I	0.18 μm CMOS RX; BCD TX	42 × 42 100 μm pitch 254 dpi	23	<21 (reported IC power)	Peak-to-peak SNR: 15.1 dB; ΔC: 50 aF; 0.3 mm cover glass.
[7] 2022/IEEE TCAS-I	0.18 μm BCD	41 × 32 150 μm pitch 169 dpi	120	23.2 (reported IC power)	SNR: 13.4 dB; resolution: 17 aF rms; 0.2 mm cover glass.
This work	0.18 μm CMOS	144 × 40 50 μm pitch 508 dpi	157.2 → 88.0 ^d^	4.03 → 7.36 ^d^ (frame- averaged)	Contrast: 5.71% → 43.86% (7.68×); image-based ridge–valley SNR > 12 dB.

Note: N/R: not reported. The arrow symbol (→) denotes the change from unit-gain mode to amplification and sensing mode for Ref. [19], and from *N* = 1 to *N* = 5 for this work. Reported metrics follow the definitions and measurement conditions of the respective publications and are not directly comparable. Power values also differ in operating mode and circuit scope; the reported IC power in [7] and [22] should not be interpreted as complete-system power. ^a^ Ref. [17] reports 1.14 fF/MPa in the main-text simulation analysis and 1.41 fF/MPa in the abstract; the main-text value is reproduced here. ^b^ Ref. [2] reports a 90 × 90 μm^2^ cell, a 100 μm pitch, and a resolution of 282.2 dpi. The reported resolution is retained. ^c^ Ref. [2] estimates the scan rate using a 1 MHz integrator clock, five integration steps, and two read phases. Its SNR gain is defined as output SNR minus input SNR. ^d^ This-work values correspond to *N* = 1–5. Frame rate is calculated as 1/*T*_capture_, and frame-averaged power as *V*_DD_ × *I*_avg_, using the measurements in Table 3. The reciprocal capture time is not an independently measured continuous-streaming frame rate. The supply voltage used for the power calculation is 3.3 V. The contrast and image-based SNR values for this work are means over the nine retained frames per accumulation number.

## Data Availability

The data presented in this study are available from the author upon reasonable request. Raw fingerprint images are not publicly available due to privacy considerations.
